# Development of Visual Motion Perception for Prospective Control: Brain and Behavioral Studies in Infants

**DOI:** 10.3389/fpsyg.2016.00100

**Published:** 2016-02-09

**Authors:** Seth B. Agyei, F. R. (Ruud) van der Weel, Audrey L. H. van der Meer

**Affiliations:** Developmental Neuroscience Laboratory, Department of Psychology, Norwegian University of Science and TechnologyTrondheim, Norway

**Keywords:** brain and behavioral development, visual motion perception, optic flow processing, perceptual information for action, prospective control

## Abstract

During infancy, smart perceptual mechanisms develop allowing infants to judge time-space motion dynamics more efficiently with age and locomotor experience. This emerging capacity may be vital to enable preparedness for upcoming events and to be able to navigate in a changing environment. Little is known about brain changes that support the development of prospective control and about processes, such as preterm birth, that may compromise it. As a function of perception of visual motion, this paper will describe behavioral and brain studies with young infants investigating the development of visual perception for prospective control. By means of the three visual motion paradigms of occlusion, looming, and optic flow, our research shows the importance of including behavioral data when studying the neural correlates of prospective control.

According to Gibson's ecological theory of visual perception, direct and precise specification of objects and events in the environment provides information for direct perception through the pattern of light reflected from the surrounding to an observer (Gibson, 1966, 1979). Integral to this theory is the concept of affordances, which refers to what the environment affords or offers the observer. For example, surfaces of the environment may afford the observer locomotion, collision with other objects, and other behaviors that may be beneficial or injurious. Thus, it is important for affordances to be perceived efficiently. According to the theory, information for visual perception is inherent in the ambient light when an observer looks at a visual scene. As such, information about the surface layout and layouts of different objects and places in the environment projects from the dynamic ambient optic array of light that reaches the eye, which then specifies action possibilities to the observer. With movement, the dynamic optic array (flow field) specifies information about direction of motion and the relative movement of objects and the observer. This pattern of visual information that results from an observer's own motion is referred to as optic flow (Gibson, 1979).

The visual motion perception that is achieved by changes in optic array information becomes crucial for environmental navigation. Optic flow patterns afford the adjustment of posture, perception of time-to-contact, avoidance of obstacles, and reaching a target efficiently by specifying the appropriate heading direction. Infants respond to radial flow patterns using defensive responses such as backward head movements and eye blinks (e.g., Kayed and van der Meer, 2000, 2007). Such responses suggest that young infants use perceptual information to execute adaptive motor responses (Shirai and Yamaguchi, 2010). In this paper, we discuss the development of the visuo-cognitive systems, especially visual motion perception for the control of anticipatory actions during early infancy. We provide information that contributes to the understanding of the development of visual motion perception for prospective control and the developmental impairments associated with motion perception following preterm birth. Understanding functional brain development and the possible developmental anomalies of premature birth is important to ensure early intervention and diagnosis of preterm infants at risk of developing neurological impairments.

## Information for prospective control

For effective navigation to reach a destination, it is vital to perceive the visual scene and then guide forthcoming actions through the coupling together of perceptual information, cognition, and the subsequent motor execution of intended actions. This ability is referred to as prospective control (Lee, 1993, 1998; von Hofsten, 1993). Prospective control is primarily concerned with future events or future goals to be realized (see also Turvey, 1992). Without sufficient prospective control, individuals may experience problems when responding to changes in the environment. Problems may include difficulties with performing everyday tasks such as the control of walking speed and direction to reach an intended destination. Controlling speed and direction during locomotion may depend on the extent of the complexity or familiarity associated with the visual flow information. As the speed of simulated forward motion increases, latencies in response to motion activity become longer (Vilhelmsen et al., 2015a). Thus, visual scenes that are perceived as being complex and naturally infrequent or unfamiliar may affect the output of cortical responses. Constant modification of integrated inputs from the visual system concerning the nature of the visual scene is therefore necessary. This modification must be dynamic enough to incorporate the constantly changing contextual information from the environment to provide accurate prospective control information.

During visually guided actions, an observer reaches an ideal state when he acts to produce a certain pattern of visual flow. This pattern is characterized by an invariant property that is left unchanged across conditions whenever the observer is in the ideal state (Fajen, 2005). Thus, when current conditions are set constant, information about one's future trajectory is used to modify deviations from the ideal state in order to eventually reach the intended outcome or destination. Over the years, models of visually guided actions for locomotion have been proposed (see Fajen, 2005). Among these models are the bearing angle model and the affordance-based model. In the bearing angle model (e.g., Lenoir et al., 1999; Fajen and Warren, 2007), an observer is on a collision course with an object if the object's bearing remains constant. Thus, to avoid collision an observer must change his speed and/or direction if there is a fixed bearing angle between the observer and the object (see also recent studies by Bootsma et al., 2015 for an extension of this model). The bearing angle model has been used by numerous studies over the years to investigate interception and detection of collisions, and obstacle avoidance in humans and other animals (see e.g., Cutting et al., 1995; Chardenon et al., 2004; Ghose et al., 2006). However, its numerous limitations (see review by Fajen, 2013) including failure to take locomotor capabilities and limits of observers into consideration, and to account for coordination of speed and direction during locomotion, have made its approach unsuitable to predict guided movement of observers in the presence of other moving objects (Fajen et al., 2013). The affordance-based model, which originates from Gibson's ecological theory, rectifies such limitations. It incorporates the ability to choose actions and guide locomotion by taking into account body dimensions and dynamics (Warren and Whang, 1987; van der Meer, 1997; Fajen, 2007, 2013). It also accounts for how speed and direction are coordinated (Warren and Rushton, 2007, 2009; Bastin et al., 2010). However, specific actions observers have to select to actualize the intended motor outcome, and the directions observers have to follow to reach their desired target fall outside the scope of what this model predicts. For successful performance during visually guided action, it is ultimately important for an observer to perceive the available possibilities for action and to behave in order to keep the desired prospective action within the range of possible actions (Fajen, 2007).

According to Gibson's ecological theory, it is important to identify stimulus variables that are necessary to specify perceived aspects of the environment. Specifying variables (optical invariants) are patterns of ambient-energy arrays that are left unchanged by certain transformations (Fajen, 2005). Tau (Lee, 1976) is an example of a specifying variable that estimates time-to-contact information for timing interceptive actions. Further studies show that an alternative source of optical information when estimating time-to-collisions is the use of non-specifying variables (e.g., visual angle and expansion rate) that do not relate to specific environmental factors (see Michaels et al., 2001; Smith et al., 2001; Jacobs and Michaels, 2006). Thus, in contrast to optical invariants such as tau that is unaffected by changes in environmental conditions, non-specifying variables are influenced by environmental factors such as speed and size of objects (Runeson and Vedeler, 1993; van der Meer et al., 1994; Fajen, 2005). Studies have shown that in estimating time-to-collision, observers may use tau information independently (e.g., Yilmaz and Warren, 1995) or in conjunction with the use of non-specifying variables (e.g., Jacobs et al., 2001; Smith et al., 2001). In this paper, studies are presented that show age-related differences in the use of specifying and non-specifying optical variables, as well as the developmental changes in the use of such variables for prospective control during perceptuo-motor tasks in infants.

Perception of visual information for locomotion includes being able to accurately time and efficiently guide movements. The introduction of the tau-coupling theory has helped to explain how organisms are able to guide their movements through the closure of motion gaps (van der Weel et al., 2007). Tau of a motion gap is the time to closure of the motion gap at its current rate (Lee, 1998). When two or more taus are coupled over a period of time, they remain in constant proportion over the specific time period (Lee, 2009). Their relationship is defined by the coupling constant, K, which defines the speed profile of the gap closure. When reaching with the hand to catch a moving object, motion gaps exist between the hand and the object, or between the hand and the estimated interception point of the object, or between the object and the interception point. For the hand to be at the correct place to catch the moving object, tau of the motion-gap between the hand and the interception point, and the tau of the motion gap between the object and the interception point must be coupled together. Thus, external information about the motion of the object tau-guides the hand in an extrinsic tau-coupling process (Lee et al., 2001). In an intrinsic tau-coupling, tau of the gap between the hand and the stationary object is performed when self-guided action is coupled with an intrinsic tau value generated in the nervous system (Lee, 2009). Tau information is in the form of electrical energy that flows in neuronal assemblies in the nervous system. Tau information in the nervous system serves as a template for movement control upon which proprioceptive feedback can be used for prospective control (Lee, 2009). Intrinsic tau-coupling activity can be observed, for example, during the control of sucking in infants where the sucking pressure follows a pressure curve predicted by tau-coupled movement (Craig and Lee, 1999), or during the control of balance in children and adults (Austad and van der Meer, 2007; Spencer and van der Meer, 2012).

## The neuronal basis of visual motion perception

In determining how visual perception is mediated in the brain, studies in humans and other primates have investigated the cerebral networks specialized for perception of visuo-spatial information over the past years. Several studies associate the structural and functional organization of the dorsal and ventral streams in the overall processing of visual information (e.g., see review by Creem and Proffitt, 2001). Perception of spatial aspects of stimuli such as the direction and speed of motion is processed via the dorsal visual stream (Creem and Proffitt, 2001), with the ventral visual stream primarily suggested to be involved in object recognition (Milner and Goodale, 2008). Neurons within the middle temporal complex (MT/V5+) of the dorsal visual stream are generally sensitive to radial motion processing including information from looming stimuli (Greenlee, 2000). The dorsal medial superior temporal (dMST) area is specifically implicated in optic flow processing (Duffy and Wurtz, 1991; Greenlee, 2000). The MT+ complex has also been found to play an important role in the control of continuous eye movement and in catch-up saccades to a moving target during the perception of motion information (Orban de Xivry and Lefèvre, 2007).

Over the years, non-invasive electroencephalogram (EEG), with its high temporal resolution in the millisecond scale, has been used to study the neuronal basis of motion perception and the functional specializations of cortical structures. EEG records brain electrical activities primarily from pyramidal neurons. In visual perception tasks, visual evoked potential (VEP) waveforms in EEG are generally assumed to represent responses of cortical neurons to changes in afferent activity (Brecelj, 2003). VEP waveforms are dominated by a motion-sensitive negativity (N2) during visual motion processing. The N2 is assumed to originate in area MT/V5, with adult N2 latencies reported around 130–150 ms (Probst et al., 1993; Heinrich et al., 2005) and around 180–220 ms in 8-month-old infants (van der Meer et al., 2008a).

Together with VEPs, EEG analysis in the time-frequency domain is used to isolate event-related frequency changes that reflect oscillatory mechanisms underlying neuronal populations (Hoechstetter et al., 2004). Event-related time-frequency responses (TSE, time spectral evolution) represent interactions of local cortical neurons that control the frequency components of an ongoing EEG (Pfurtscheller and Lopes da Silva, 1999). Using spectral profiles within specific frequency bands, different classes of oscillations have been distinguished over the years: delta-band (1–4 Hz), theta-band (4–7 Hz), alpha-band (7–13 Hz), beta-band (13–30 Hz), and gamma-bands (30–150 Hz). These rhythms are thought to reflect neurophysiological processes that exhibit functionally different roles. These roles include signal detection and decision making with the use of delta frequency (Başar et al., 2000), the control of inhibition and cortical processing with alpha-band waves (Klimesch et al., 2007), involvement in multisensory stimulation and the shifting of neural systems to a state of attention using beta-band activity (Khader et al., 2010), and the utilization of bottom-up and top-down memory matching of information for perception using gamma frequency (Herrmann et al., 2010). Several adult studies have found evidence for the modulation of the natural frequencies by motion stimuli (e.g., see review by Saby and Marshall, 2012), with little evidence for such activity reported in infants. Low-frequency EEG rhythms are reported in infants (e.g., Orekhova et al., 2006), with event-related theta oscillations found to provide information for impending collisions in the infant brain (van der Weel and van der Meer, 2009). Some of the studies presented in this paper will show further evidence for the use of theta-alpha and other frequency oscillations during the processing of visual information for the control of prospective actions in infants.

## Early development of visual perception for prospective control

Since perception of information for prospective control plays an important role for everyday survival, the developmental processes that mediate visual perception throughout life are expected to be increasingly efficient after birth. One of the earliest indicators of prospective control behavior in infants is the ability to continuously pursue a moving target with head and eye movements (von Hofsten and Rosander, 1996). Smooth visual pursuit of a moving target involves fixing gaze on the target and matching eye movements with the speed of the moving target. This helps to anticipate and predict the target's trajectory. Rudimentary perception of visual flow appears within the first weeks after birth (Shirai and Yamaguchi, 2010). Infants younger than 6–8 weeks are unable to efficiently discriminate between motion directions or smoothly pursue small moving objects, but they show rapid improvements between 6 and 14 weeks of age (Gilmore et al., 2007; Rosander et al., 2007). Young infants exhibit sensitivity to information for impending collision very early in development, with infants between 3 and 6 weeks shown to perceive optical collisions by responding with defensive blinks and head movements (e.g., Náñez, 1988). Even neonates as young as 3 days old exhibit responses through backward head movements when exposed to backwards flow stimuli (Jouen et al., 2000; Shirai and Yamaguchi, 2010). Such responses in very young infants may be the result of multimodal integrative and cooperative processes in which visual, vestibular, and proprioceptive senses are involved rather than a direct consequence of motion perception (Jouen et al., 2000).

Around 2 months of age, infants are already able to show prospective control as they continuously track objects using smooth pursuit eye movements and a gain geared to the velocity of the moving target (Rosander and von Hofsten, 2002). From 3 to 5 months, infants discriminate between virtual flow displays that depict at least 22° changes in heading direction (Gilmore et al., 2004). Around 6 months of age, they further follow moving objects on a linear path using predictive head and eye movements (Jonsson and von Hofsten, 2003). At this age, infants reach for a moving target by not aiming for the current position of the object but predictively aiming for a position further ahead on the path where the hand and the object will meet (van der Meer et al., 1994; von Hofsten et al., 1998; Jonsson and von Hofsten, 2003). When moving objects that are being tracked move temporarily out of view, infants should anticipate where and when the object would reappear again. This ability seems to be developed around 6 months of age (Johnson et al., 2003).

Studies using anticipatory and compensatory postural adjustment to study prospective control have found mobile infants around the end of the first year of life to show peak postural compensation to visual flow information (e.g., Bertenthal et al., 1997; Lejeune et al., 2006). Witherington et al. (2002) studied infants between 10 and 17 months of age to investigate early development of anticipatory postural activity in support of pulling action. Infants retrieved toys by pulling open cabinet drawers while a force resisting the pulling action was applied to the drawers. Infants' anticipatory postural adjustments and the temporal specificity of anticipatory activities progressively improved with age as infants learned to stand and walk. By improving anticipatory postural responses, balance control is also enhanced (Santos et al., 2010). Thus, prospective control plays an important role in keeping balance during standing and locomotion. In evaluating whether infants who are able to walk show greater sophistication compared to non-walking infants when anticipating postural disturbances induced by a continuously moving platform, Cignetti et al. (2013) reported that the acquisition of independent walking improves sensorimotor control of posture. Other studies also show that infants with locomotor experience typically respond more to peripheral flow than pre-locomotor infants and that the developmental shift in using flow-field information for postural control may be more closely linked to locomotor experience (e.g., Higgins et al., 1996). With the development of self-generated actions including self-locomotion experience, what is perceived and the ensuing anticipatory actions considerably improve in the developing brain (van der Meer et al., 2008a; James and Swain, 2011). Thus, the functional detection of visual flow information develops hand in hand with self-produced locomotion in normally developing infants (van der Meer et al., 2008a).

Unlike normally developing full-term infants, preterm infants show differential brain development that is particularly evident from abnormalities in tissue microstructure, cerebral morphology, and white matter damage (see review by Counsell and Boardman, 2005). Preterm infants are therefore at a higher risk of developing neurological and perceptuo-motor problems (see Taylor et al., 2009). These abnormalities underlie various cognitive and behavioral impairments, including deficits in visual perception and other neurodevelopmental disorders that are associated with preterm birth (de Jong et al., 2012). Preterm children show deficits in perception of global motion, global form, and biological motion, with impairment of the dorsal visual stream particularly implicated as a possible cause of such developmental problems (Taylor et al., 2009). Because of these impairments, identifying at-risk preterm infants is necessary to offer appropriate early intervention to those who need it.

By using brain and behavioral data mainly from occlusion, looming, and optic flow studies, we further discuss the development of visual perception for the control of prospective actions during the first year of life. Prospective control behavior in infants is shown through predictive gaze and reaching movements, and different timing strategies for obstacle avoidance. We show that the development of prospective control substantially improves with age. We illustrate how preterm infants show developmental delays in the processing of prospective control information by comparing full-term infants' responses with responses of preterm infants. The relationship between behavioral development and the development of the underlying neuronal processes is highlighted through EEG measurements of neuronal electrical activity as a function of perception of visual motion information.

## Interception tasks with temporary occlusion

With visual occlusion tasks, we investigated infants' prospective control and the ability to maintain object permanence—the understanding that an object exists even if it is out of sight. The development of the mediating neural structures of such processes was also studied. By combining behavioral measurements of eye, head, and hand-reaching movements together with EEG analysis of neuronal gamma oscillations, we could study infants' ability to follow, maintain attention on, and predict the arrival of a moving object as it disappears behind an occluder and reappears shortly afterwards.

When reaching for a moving object, infants must use prospective control to guide their hand-reaching movements to intercept the moving object. To study prospective control in catching, van der Meer et al. (1994) investigated the control of hand and gaze movements as infants reached for a toy moving at different speeds. The toy was occluded from view by a screen during the final part of its approach. Infants could reach to catch the toy when it was at a certain distance or time away from them. To effectively catch the toy, a strategy based on distance is less efficient as it is dependent on the approach velocity of the toy. Thus, reaching to catch the toy when it is approaching at a fast velocity leaves very limited time to extend the arm to make an interceptive movement. A strategy based on time-to-contact, however, is most efficient since it leaves the same amount of time to carry out the interceptive movement irrespective of the toy's approach velocity. Infants around 11 months of age anticipated with their gaze and hand the reappearance of the toy as it emerged from behind the occluder. Their hands started moving forward before the toy had even disappeared behind the occluder in order to catch the toy as soon as it reappeared. Prospective gaze and hand action was coupled to certain times before the toy's reappearance. Thus, information that was picked up before the disappearance of the toy behind the occluder was used to regulate gaze and hand movement. When infants between 20 and 48 weeks of age were studied longitudinally, infants' gaze anticipated the reappearance of the moving toy as soon as they were able to successfully catch the toy. Infants' anticipatory gaze movements suggest that this ability is a prerequisite for the onset of reaching for moving objects. As corroborated by various studies (e.g., Aguiar and Baillargeon, 1999), the findings indicate that object permanence is present in infants earlier than the suggested 8 months by Piaget (1954). The ability to successfully catch a fast-moving object coincides with infants' ability to use perceptual information to initiate a reaching movement. Initiation of the hand movement should begin when the toy is a certain time away from them, instead of a certain distance away, thus making available the same average time for the catching movement whether the toy is moving slowly or quickly.

How do neurologically at-risk preterm infants perform in comparison with full-term infants on tasks that rely heavily on prospective control? van der Meer et al. (1995) studied healthy full-term infants and low-birthweight preterm infants longitudinally between 20 and 48 weeks of age to investigate whether infants classified as being neurologically at-risk of brain damage have similar prospective control ability as full-term infants, and if not, whether their lowered ability could be indicative of brain damage. Infants' ability to reach for a toy moving at different speeds was studied. At the first reaching session each infant's gaze successfully anticipated the reappearance of the moving toy. However, reaching onset and prospective control of gaze and hand movements varied considerably between the full-term and preterm infants. From 24 weeks onwards, the full-term infants anticipated the moving toy with their gaze, but gaze anticipation was delayed in all the preterm infants until 40 or 48 weeks of age. As a group, the preterm infants started reaching late for the toy. Three started to reach at 28 weeks corrected age, 8 weeks later than the full-term control infants. Some preterm infants geared their actions to the distance instead of the time that the toy was from the catching point, which caused problems with faster moving toys. Almost all the preterm infants anticipated the reappearance of the moving toy with their hand at the final testing session at 48 weeks of age. They also started showing signs of using the time strategy to adapt their actions according to the length of time that the toy was from the reappearance point at this age. Two of the preterm infants still appeared to be using the less efficient distance strategy when shifting their gaze and initiating their hand movement to reach for the toy at 48 weeks of age. The same two infants also showed the poorest anticipation of the toy's reappearance. These two preterm infants were later diagnosed with mild and moderate cerebral palsy at around 2 years of age. Hence, poor development of prospective control on the catching task could potentially serve as an indicator of possible brain damage.

Further, with normally developing full-term and preterm infants between 22 and 48 weeks of age, we longitudinally investigated the timing strategy infants use to initiate and guide the hand when catching a moving object and whether the guiding action is influenced by the use of timing strategies (Kayed and van der Meer, 2009). Little difference was found between full-term and preterm infants' use of timing strategies. Preterm infants showed about the same development as full-term infants both in timing the catch and in continuously guiding hand movement. Variation in the functionality and length of the tau-coupling between the hand and the toy was influenced by the timing strategy the infants were using to initiate the hand movement. The younger preterm and full-term infants used a distance strategy to initiate hand movement when they started to reach for the moving toy. This resulted in a high number of unsuccessful attempts at catching the toy. They performed shorter and less functional tau-coupling that was characterized by non-controlled collisions with the hand accelerating toward the toy when they used the distance strategy. However, the older infants around the end of the first year of life switched to a time strategy when reaching for the moving toy. They performed longer and more functional tau-coupling between the hand and the toy, with better controlled collisions with the hand decelerating toward the toy. They showed a marked improvement in the number of successful catches. One preterm infant failed to switch to a time strategy and showed poor prospective control with a higher number of unsuccessful catches compared to other infants. This preterm infant may later have perceptuo-motor problems.

To further investigate the use of prospective control in catching and how it could be used as a tool to detect signs of brain dysfunction, Aanondsen et al. (2007) studied adolescents between 14 and 15 years of age who were either born as preterm very-low-birthweight (VLBW), full-term small for gestational age (SGA), or full-term appropriate for gestational age (AGA) infants. They were presented with a moving target that approached from the side at three different accelerations. The experiment was conducted as a blind study without knowing beforehand the participants' neurological status such as birth status, gestational age, birthweight, and their cerebral magnetic resonance imaging (MRI) results. All participants used the time-to-contact strategy to initiate their hand movements except three adolescents (two preterm VLBW and one full-term SGA). They rather used the less advanced distance or velocity timing strategy to guide the initiation of at least one of their hands to catch the moving target. Based on their timing strategies, the three adolescents were classified as at risk for neurological problems. Their cerebral MRI confirmed this classification. It showed them to have reduced white matter tissue, dilation of the ventricular system, and/or pathology in the corpus callosum. The findings showed that the ability to use prospective information for catching could be a reliable tool to help detect diffuse signs of motor dysfunction that may not be readily detectable using only standard neuropsychological tests.

**Figure 1:**
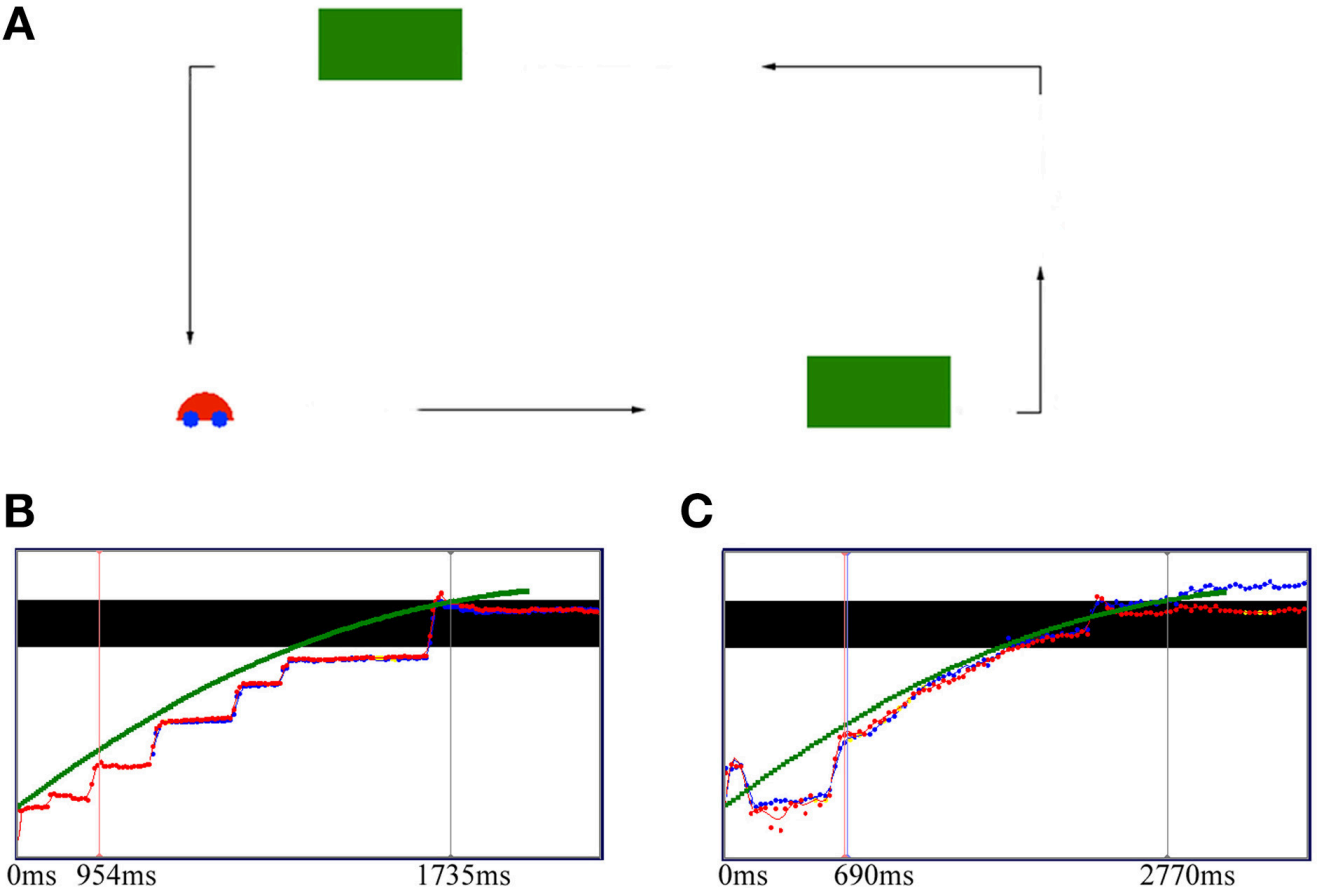
**Occlusion experimental set-up (A), and gaze data (↑ position and → time) for a typical slow deceleration in a 4-month-old infant (B) and a 12-month-old infant (C)**. **(A)** The car traveled horizontally on a rectangular path under one of three deceleration conditions, fast (10% deceleration), medium (50% deceleration), and slow (90% deceleration). The two green boxes temporarily occluded the car from its path of travel. **(B,C)** The black bar represents the occluder, and the green squares represent the car motion, while the red and blue dots represent the right and the left eye, respectively. Yellow dots are missing data. The left markers in each graph represent the catch up event (the moment at which the horizontal eye velocity equals the speed of the car for the first time), while the right markers represent the point in time at which the car starts to reappear from behind the occluder. The 4-month-old infant shows typical saccadic tracking to keep up with the target motion **(B)**, whereas the 12-month-old infant follows the car with smooth pursuit **(C)**. The 12-month-old infant shows an anticipatory saccade to the end of the occluder before car reappearance, while the 4-month-old infant shows no such prospective eye movement.

To investigate the neural correlates underlying prospective control, Holth et al. (2013) coupled adults' gaze control during deceleration in a visual tracking task with their EEG activity. Participants followed with their gaze a horizontally moving car that was temporally occluded and pushed a button to stop the car as soon as it reappeared from behind the occluder on a large screen placed 80 cm in front of them (see Figure 1A). The car moved under three different constant decelerations. The button-press response was defined as either a hit or a miss depending on how much of the car was visible in the target area when it was stopped. A hit response was defined as at least half of the car being visible after pressing the button. Different events were used to time-lock the averaged event-related potential (ERP) waveforms, including stimulus onset, push-button responses, and eye jumps across the occluder. When ERP waveforms were time-locked to the prospective gaze shift over the occluder, participants were successful in discriminating between the three decelerating speeds. Thus, participants' parietal activity indicated that they were able to differentiate between the different car decelerations but only when their averaged EEG was time-locked to the eye jump event and only when they managed to stop the car successfully. No such effect was found when ERP waveforms were time-locked to any of the other events. The findings indicate that a traditional stimulus-onset time-locking procedure is likely to distort the averaged EEG signal. This distortion may consequently hide important activity differences, especially in the parietal cortex that may provide information about the prospective timing of decelerating object motion during occlusion. The observations strongly suggest active incorporation of behavioral data into EEG analysis to provide valuable information that would be lost otherwise, when studying the neural correlates of prospective control.

Further longitudinal EEG studies showed that infants' ability to smoothly track a moving object undergoing occlusion (see Figure 1A) and to predict its reappearance increases considerably between the ages of 4 and 12 months (Twenhöfel et al., 2013). Infants showed more instances of shifting gaze predictively over the occluder with age (Figures 1B,C). The older infants showed a more consistent pattern of anticipatory eye movements in response to the moving target. The results corroborate previous studies showing that anticipatory eye movements improve considerably in the course of the first year of life (see e.g., Gredebäck and von Hofsten, 2004). In order to successfully track an object over an occlusion period, object permanence must be developed. Rosander and von Hofsten (2004) suggested that smooth pursuit of moving targets and predictive occluder tracking depend on the ability to anticipate future motion based on the prediction of a continuous motion trajectory of a moving object. Because of a 100–200 ms visuo-motor delay that the smooth pursuit system has to overcome during the tracking of moving objects (see Schlag and Schlag-Rey, 2002), smooth pursuit must be adjusted predictively to compensate for this delay within which a visual target may have moved significantly. With the development of object permanence, the older infants may have used visuo-motor integration to successfully predict the object's trajectory and to continuously track its movement.

The predictive gaze shift was accompanied by a divergence and a shift in gamma band topography with age. Neuronal gamma band topography shifted from occipital areas in the dorsal stream in the younger infants to anterior temporal areas in the ventral stream in the older infants when the underlying neuronal source activities were analyzed. The divergence in gamma band topography may possibly reflect developmental changes in neuronal mechanisms serving object tracking over transient occlusion periods during the course of the first year of life. Previous studies have also implicated gamma activity in complex object processing in regions distributed along the ventral and dorsal pathways (e.g., Lachaux et al., 2005; Hoogenboom et al., 2006). The shift of gamma activity in neuronal regions may suggest different strategies of occluder tracking with age. Younger infants may be guided mainly using spatio-temporal information processed via the dorsal pathway to fill perceptual gaps over transient occlusions. The ventral pathway activation in the older infants may suggest further incorporation of object features during perceptual representations of moving objects. Thus, the gamma activation could represent top-down processing (high-speed memory comparison) of the object template that was maintained over the perceptual gap with the perceived stimulus (see Herrmann and Mecklinger, 2001). The ventral stream activation is in accordance with the suggestion that vision for perception (a typical ventral stream task) could replace vision for action (mainly a dorsal stream task) in order to successfully guide 11-month-old infants' arm reaching movements in an occlusion situation (van Wermeskerken et al., 2011). The developmental progression in regional cortical shift of oscillatory activity suggests that the development of object permanence and prospective control become more prominent around the end of the first year of life.

Unlike full-term infants, preterm infants show delayed development in the continuous eye tracking of moving objects. While full-term infants around 12 months smoothly followed the moving target in 64% of all trials, preterm infants around the same age (corrected for prematurity) showed smooth pursuit in only 35% of the presented trials. The lower proportion of predictive eye movements in the preterm infants compared to the full-term infants may be a reflection of a weak object representation (Munakata, 2001) and a delay in the influence of functional object representations on eye movements (Hollingworth et al., 2008). However, their ability to make anticipatory eye movements was relatively similar to the full-term infants. Thus, they were able to disengage attention from tracking the moving object during an occlusion period and then predictively re-orient gaze over the occluder after the object's reappearance despite showing difficulties with smooth pursuit. Disturbances in the development of the motion perception pathways and other complications associated with premature birth may impair motion processing and contribute to preterm infants' reduced ability to track moving objects. To compensate for their less functioning smooth pursuit system, it has been suggested that preterm infants may use saccadic eye movements and head movements to continuously follow a moving target, although this results in less efficient smooth pursuit than that observed in full-term infants (Grönqvist et al., 2011).

## Looming virtual stimuli on a collision course

How does the infant brain process information about imminent collisions? By simulating a looming object on a direct collision course toward infants, it is possible to investigate brain activities in response to looming information. Looming refers to the last part of the approach of an object that is accelerating toward the infant (Kayed and van der Meer, 2007). To prevent an impending collision with the looming object, infants must use a timing strategy that ensures they have enough time to estimate when the object is about to hit them in order to perform the appropriate behavioral response. Defensive blinking is widely considered as an indicator for sensitivity to information about looming objects on a collision course. Infants must use time-to-collision information to precisely time a blinking response so that they do not blink too early and reopen their eyes before the object makes contact or blink too late when the object may have already made contact. An accurate defensive response helps to prevent injury to the infants. For a successful defensive response to avoid collisions, development of prospective control is important. Infants must use looming visual information to correctly time anticipatory responses to avoid impending collisions.

The timing strategies that infants use to determine when to make a defensive blink to a looming virtual object on a collision course were investigated using full-term infants between 22 and 30 weeks of age in a cross-sectional behavioral study (Kayed and van der Meer, 2000). The youngest infants used a strategy based on visual angle (analogous to the distance strategy) to time defensive blinks. Thus, they blinked too late when the looming object approached at high accelerations. The oldest infants, on the other hand, used a time strategy allowing them to blink in time for all the approach conditions of the virtual object. When precise timing is required, the use of the less advantageous visual-angle strategy may lead to errors in performance compared to the use of a time strategy that allows for successful performance irrespective of object size and speed.

Further longitudinal studies of full-term and preterm infants at 22 and 30 weeks of aged showed that with age, the majority of infants switched from using a strategy based on visual angle to a strategy based on time to time their blinks (Kayed and van der Meer, 2007; Kayed et al., 2008). Some of the infants used a time strategy even already at 22 weeks, with such infants maintaining the use of this strategy on subsequent testing sessions. None of the infants switched back to using a strategy based on visual angle after using a time strategy. One preterm infant showed delayed development compared to the other infants since he was using a timing strategy based on visual angle for all loom speeds. This caused him to blink late on the majority of trials even when he was 30 weeks of age. In infants, the inability to switch from a timing strategy that is susceptible to errors to a strategy that affords successful defensive blinking might reflect an inadequate potential for flexibility. Flexibility may be required to help adjust appropriately to local environmental conditions and to successfully interact with the environment, especially since good timing is essential to avoid obstacles during navigation.

**Figure 2:**
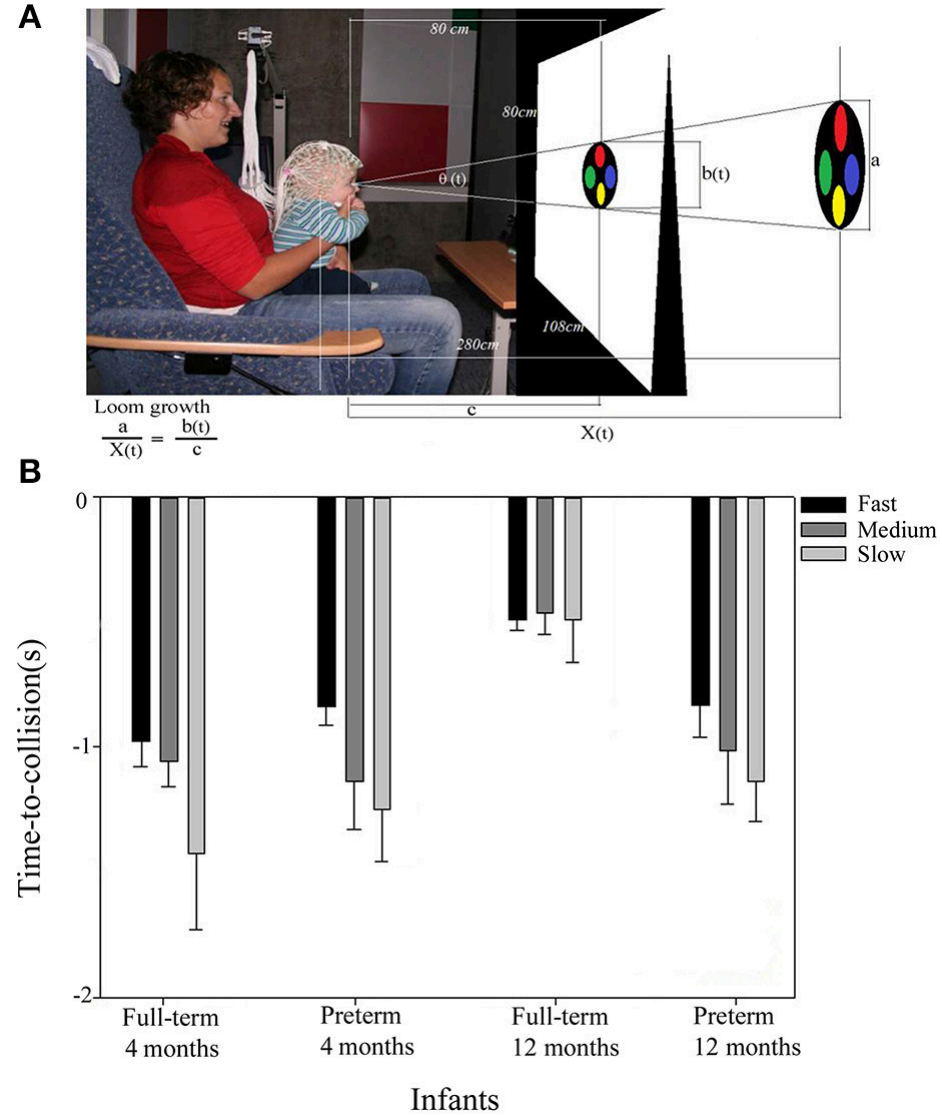
**Looming experimental setup (A), and averaged looming-related VEP peak responses (with SDs) in full-term and preterm infants (B)**. **(A)** Infants were shown a flat 2-dimensional circle filled with four smaller colored circles. The looming stimuli simulated an object approaching from a distance on a direct collision course under constant accelerations of −21.1, −9.4, −5.3 ms^−2^ for fast loom (2 s), medium loom (3 s), and slow loom (4 s), respectively. The bottom left equation describes the growth of the visual loom. The looming stimuli approached the infant as the image on the screen grew symmetrically and stopped when the image filled the entire screen. **(B)** With increasing age, the full-term infants responded significantly closer to the loom's time-to-collision compared to the pre-term infants. Only the older full-term infants responded at a fixed time-to-collision irrespective of loom speed, an indication that only the full-term infants at 12 months had switched from a visual angle strategy to the more sophisticated time strategy when timing their looming-related VEP peak responses.

With the presentation of a looming virtual object on a direct collision course, we then investigated the developmental differences between full-term and preterm infants using high-density EEG. Infants were studied longitudinally at 4 and 12 months. The looming stimulus was programmed to loom toward the infant with different accelerations, which finally came up to the infant's face to simulate a visual collision experience (see Figure 2A). Looming-related peak VEP responses were analyzed using source dipoles in occipital areas. Results showed a developmental trend in the prediction of an object's time-to-collision in full-term infants. With age, average VEP duration (processing time) in full-term infants decreased, with peak VEP response closer to the loom's time-to-collision (van der Weel and van der Meer, 2009; van der Meer et al., 2012). Full-term infants around 12 months of age used the more sophisticated and efficient time strategy to time their brain responses to the virtual collision. Their looming-related brain responses were fixed at a constant time-to-collision irrespective of visual loom speed (Figure 2B), an indication of the development of prospective control at this age (van der Meer et al., 2015). The use of such a timing strategy based on a fixed time-to-collision may reflect infants' levels of neural maturity and locomotion experience. Maturity and experience are important factors needed for accurate timing of prospective actions in response to looming objects to ensure successful evasive maneuvers during navigation.

However, unlike full-term infants, preterm infants did not show such improvements with age but continued to use the less efficient timing strategy based on the loom's visual angle even at 12 months (Figure 2B). This suggested that preterm infants have problems with prospective control during the first year of life, showing their brain responses too early in the looming sequence and therefore not adequately taking into account the loom's different accelerations.

By localizing brain source activity for looming stimuli approaching at different speeds and using extrinsic tau-coupling analysis, the temporal dynamics of post-synaptic neuronal activity in the first year of life was further investigated (van der Weel and van der Meer, 2009). Tau-coupling analysis calculated tau of the peak-to-peak source waveform activity and the corresponding tau of the loom speeds. Source dipoles that modeled brain activities within the visual areas of interest, O1, Oz, and O2 were fitted around peak looming VEP activity to give a direct measure of brain source activities on a trial-by-trial basis. Using full-term pre-locomotor infants at 5–7 and 8–9 months and crawling infants at 10–11 months of age, synchronized theta-band activity in response to the looming stimulus was found. This was consistent with other studies that identified oscillations in the theta range as important for registration and processing of visual perceptual information (e.g., Kahana et al., 2001). Extrinsic tau-coupling analysis on the source waveform activities showed evidence of strong and long tau-coupling in all infants. The oldest infants showed brain activity with a temporal structure that was consistent with the temporal structure present in the visual looming stimuli. Thus, in the course of development, the temporal structure of different looming stimuli may be sustained during processing in the more mature infant brain. Sustaining the temporal structure may provide increasingly accurate time-to-collision information about looming danger as infants become more mobile with age. Infants at 10–11 months differentiated well between the different loom speeds with increasing values of the tau-coupling constant, K, for the faster loom. The younger infants were not able to differentiate between the looms, with the worst performance observed in infants at 5–7 months. The findings may suggest mature neural networks for processing impending collision information in the oldest infants compared to the youngest. At 5–7 months, such neural networks may not have been developed but could rather be in the process of being established at 8–9 months of age, which coincides with the onset of crawling in infants. Thus, with better control of self-produced locomotion, the perceptual ability to recognize looming danger, and perform the necessary prospective action to avoid impending collision markedly improves.

In the developing brain, not only is visual information important for the performance of prospective actions, but also integration of information from multiple senses is necessary and fundamental to perception. To investigate whether the auditory system also plays a role in prospective control, van der Meer et al. (2008b) used an auditory-guided rotation paradigm in a behavioral study of infants at 6–9 months of age. Infants lay in a prone position with magnetic sensors fastened to their head and body to measure direction and velocity of rotation as they responded to auditory stimulation from their mothers. Infants were able to consistently choose the shortest way over the longest way to rotate to their mothers who were positioned behind them. The infants showed prospective control by rotating with a higher peak velocity as the angle to be covered between themselves and their mother's position increased. In line with affordance theory, we showed that the auditory system can function as a functional listening system. Auditory information may be used as a source of perceptual information to help guide behaviors adequately in the environment. Mobile infants may use auditory information that offers them the most efficient method for action relative to their own position in space and a desired position to reach in the environment (also see Morrongiello, 1988; Middlebrooks and Green, 1991; van der Meer and van der Weel, 2011).

However, when visual and auditory looming information are simultaneously present in an audiovisual looming stimulus, prelocomotor full-term infants show earlier looming-related brain responses to the auditory loom than to the visual loom (Agboada et al., 2015). Longitudinal studies show that peak visual and auditory looming activation responses in infants at 3–4 months occur earlier in the looming sequence compared to older infants at 9–10 months. The results indicate a developmental trend in the prediction of time-to-collision information in infancy where the recruitment of neuronal assemblies in higher cortical areas, particularly in the parietal cortex, is implicated in the processing of looming-related information as infants age. With an evolutionary bias for survival prioritizing an early auditory response over that of visual response in audiovisual looming perception, it is likely that audiovisual integration in infants could be heavily influenced by their spatial attention being captured by a visual loom. In order words, visual looming-related responses that appear relatively late in a looming sequence could be a reflection of infants' active attention shown to a visual loom over that of an auditory loom (see Corbetta et al., 1990).

## Optic flow information simulating self-motion

With an optic flow paradigm, we have explored the development of visual motion perception during the first year of life by using both evoked (VEP) and induced (time-spectral evolution, TSE) brain responses to simulated self-motion. Using EEG in 8-month-old infants and adults, van der Meer et al. (2008a) studied brain electrical activity as a function of perception of structured optic flow and random visual motion. Brain activities related to the processing of motion stimuli were different in infants and adults both in VEP and induced activities of EEG. Adults and infants had shorter N2 latencies for structured optic flow than random visual motion. Infants showed longer latencies in both motion conditions compared to adults, with the longest latencies observed for random visual motion. While infants used the slower theta-band frequency during the processing of visual information, adults used the faster beta-band activity in response to the motion conditions. The findings show that infants that are not yet capable of walking may detect optic flow less efficiently compared to adults and they may be more affected by the lack of structure present in random visual motion. When the speed of structured forward optic flow information was varied in adults and infants at 4–5 and 8–10 months, Vilhelmsen et al. (2015a,b) showed that differences in N2 peak latency occurred in the adults and the older infants but not in the infants at 4–5 months. N2 latencies were found to decrease with age, with shortest N2 latency observed for the lowest speed of motion. Unlike the younger infants, the older infants may have had a more developed neurobiological system that contributed to an improved detection of visual motion, similar to the adult participants. Motion-sensitive cortical areas continue to develop through infancy to adulthood (Gilmore et al., 2007), which lead to more efficient processing of different speeds of motion with age.

In relating behavioral changes such as locomotion experience to accompanying changes in brain activities, prelocomotor infants at 3–4 months and infants at 11–12 months with self-produced locomotion experience were longitudinally studied using an optic flow paradigm (Agyei et al., 2015, 2016). Both full-term and preterm infants were studied to investigate the effect of prematurity on the processing of optic flow information. The infants were presented with three motion conditions (forwards and reversed optic flow, and random visual motion) together with a static non-flow condition. The younger infants had no crawling experience while the older infants had on average, about 2.5 months of crawling experience.

**Figure 3:**
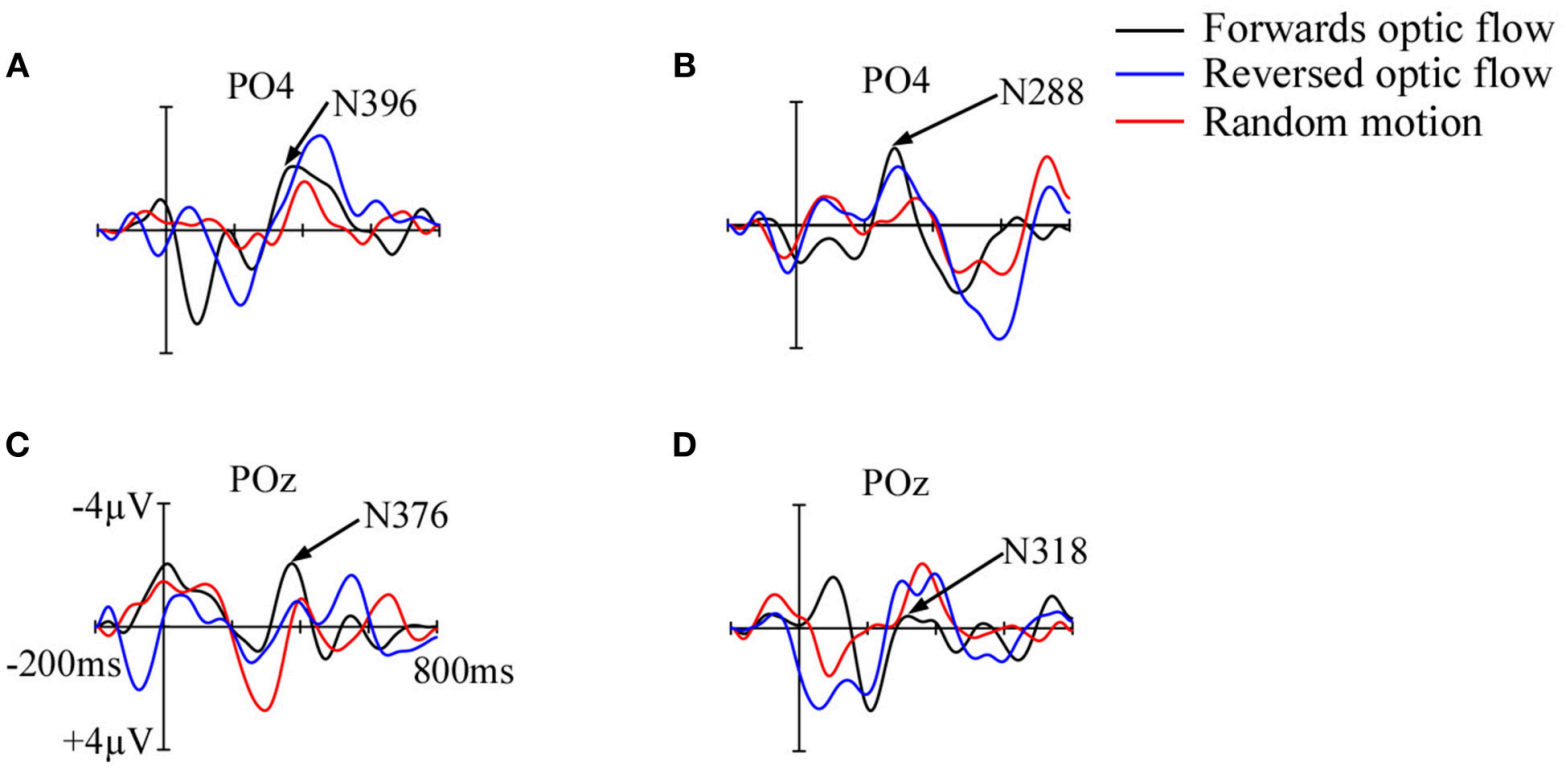
**Grand average motion VEPs in full-term infants at 3–4 months (A) and 11–12 months (B), and in preterm infants at 3–4 months (C) and 11–12 months (D)**. Amplitudes are on the y-axis and latencies on the x-axis. The actual N2 peak latencies for forwards optic flow are indicated at PO4 in full-term infants and POz in preterm infants. Differences in N2 peak latencies for the three motion conditions were observed only in full-term infants at 12 months where latency increased from forwards optic flow to reversed optic flow and random motion.

Full-term infants differentiated between the three motion conditions with shortest latency for forwards optic flow and longest latency for random visual motion, but only at 11–12 months (Figure 3). This improvement in visual motion perception with age was possibly due to significant neural developments such as increasing myelination of connecting fibers (Paus et al., 2001; Grieve et al., 2003; Loenneker et al., 2011) and maturation of local glucose metabolic rates (Chugani et al., 1996; Klaver et al., 2011). Thus, rapid progressive improvement in the functional processing of motion information as infants get older may account for the shorter latencies observed in infants at 11–12 months. The shortest latency for forwards optic flow could suggest faster sensitivity development to radial motion that corresponds to forward movement rather than to reversed or random directions. Further, when mothers carry infants, the infants experience passive locomotion where they are tuned to the dominant statistics of their experienced visual environment (Raudies et al., 2012; Raudies and Gilmore, 2014). Infants' passive experience of visual flow, especially during fast flow speeds, occurs as a result of their downward head direction and closer proximity to ground surfaces when being carried (Raudies et al., 2012). However, only self-generated actions may lead to a stronger link between perception and action in the developing brain (James and Swain, 2011). Thus, only the older full-term infants who had crawling experience from self-movement were better at distinguishing between the motion conditions compared to the younger infants who only had passive locomotion experience from being carried around.

The preterm infants did not differentiate between the three motion conditions at 11–12 months or improve their latencies with age. Studies show that preterm infants at corrected age of 2–3 months are delayed several weeks compared to full-term infants when differentiating between changes of direction (e.g., Braddick et al., 2005; Birtles et al., 2007). Considering that the preterm infants had similar crawling experience as the full-term infants, their inability to differentiate between the motion conditions when older could have resulted from abnormalities in white matter that may underlie impairment of the dorsal visual stream. Thus, axonal electrical impulses could be impaired, resulting in unimproved latencies with age. It is possible that preterm infants' unimproved latencies with age could also reflect a normal delay related to premature birth that could be recovered at a later age. However, at 3–4 months and irrespective of visual motion condition, preterm infants had significantly shorter latencies than full-term infants. Since the preterm infants were tested corrected for prematurity, one contributing factor to this faster perceptual response could be the longer exposure to and experience of visual flow in the younger preterm infants compared to the term infants at 3–4 months.

**Figure 4:**
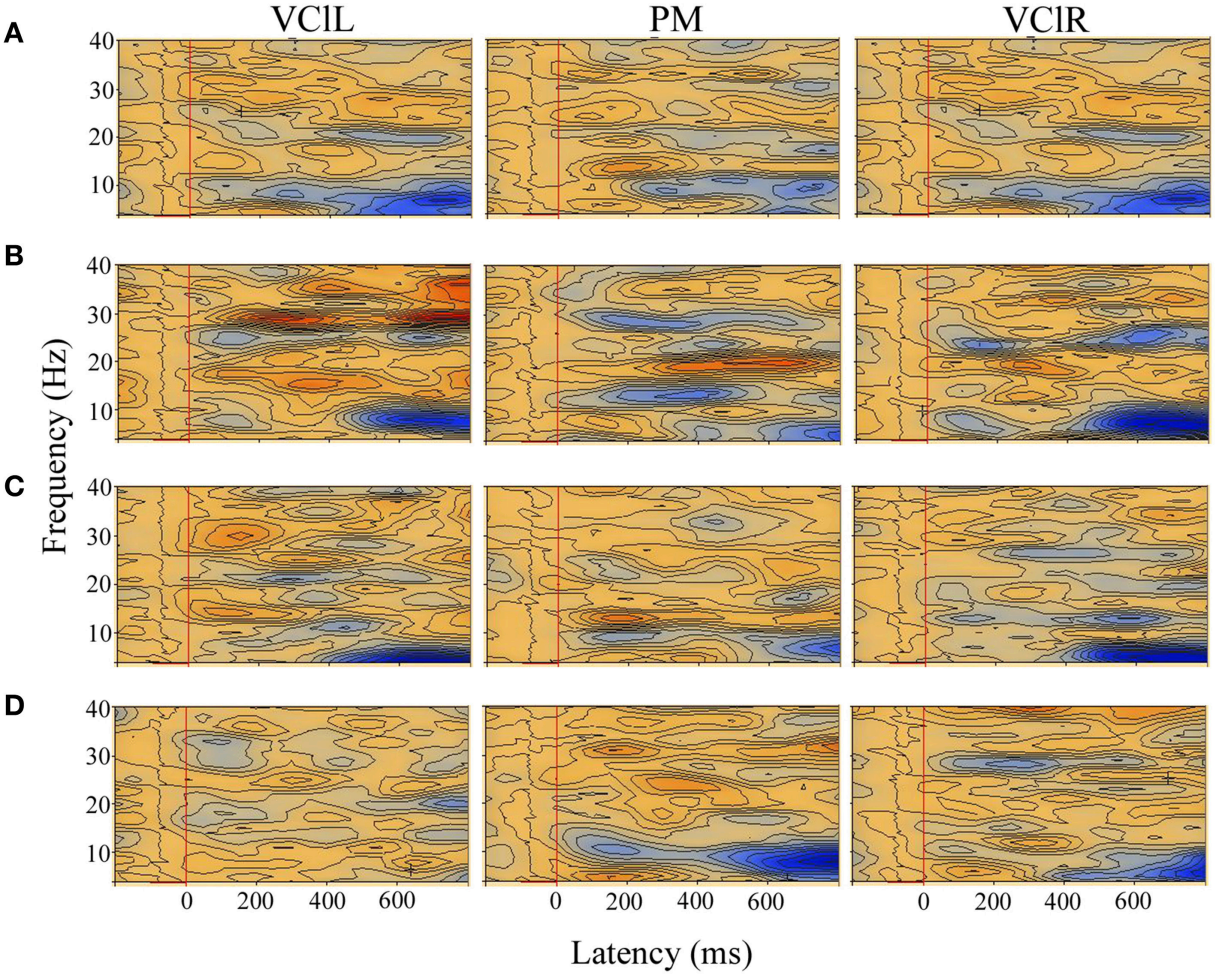
**TSE plots across brain regions of interest (VClL, visual cortex lateral left; PM, parietal midline; VClR, visual cortex lateral right) when the motion conditions were compared with the static non-flow condition in a typical full-term infant at 4 months (A) and 12 months (B), and in a typical preterm infant at 4 months (C) and 12 months (D)**. Induced synchronized and desynchronized activities appear in red and blue colored contours, respectively. Induced theta-band desynchronized activities were observed in all the visual areas of interest in the full-term and preterm infants at both ages, with induced alpha-beta band synchronized activities observed in two or more visual areas only in the full-term infants at 12 months. Stimulus onset is the vertical red line at 0 ms, with epoch from −200 to 800 ms.

When TSE of the motion conditions were compared with TSE of the static non-flow dot pattern, both infant groups showed desynchronized theta-band activity that was more prevalent in the younger infants (Figure 4). Low-frequency theta-band oscillation is a general sign of immaturity in infancy (e.g., Orekhova et al., 2006). The more prevalent theta-band desynchronization in the younger infants could suggest relatively larger neural networks and lesser specialization when processing radial motion information at this age. Further, synchronized alpha-beta band activity was seen only in the full-term infants at 11–12 months. The emergence of faster alpha-beta band frequency activity only at 11–12 months could indicate a gradual progression from less specialized, slower oscillating, and relatively immature larger oscillatory cell assemblies at 3–4 months to a more adult-like pattern of motion specialization where cell assemblies have fewer but more specialized neurons. This could explain why full-term infants at 11–12 months are better at establishing more rapid coupling between spatially separated brain regions, allowing for improved visual motion perception.

The possible impairment of the dorsal stream responsible for processing visual motion could be the reason why the preterm infants at 11–12 months showed no such progression in oscillatory patterns. Since the dorsal visual stream develops and matures relatively early (Hammarrenger et al., 2007), being born preterm may have disrupted the association fibers and synaptic development in the dorsal stream that help to fine-tune cortical growth during late fetal and early extrauterine life (Huppi et al., 1998; Mewes et al., 2006). The disruption in the development of the dorsal visual stream because of premature birth may have impeded efficient cortical growth and contributed to the absence of higher frequency oscillatory activities when the preterm infants were older. Further, individual analysis showed abnormally high latencies in response to optic flow in three preterm infants (see also van der Meer et al., 2015). Because of the possible greater degree of impairment of the dorsal stream in these preterm infants, a follow-up study when the preterm infants reach school age is necessary to investigate whether these infants still have impaired dorsal stream-related functions, and the effect of the impairment on everyday life.

## Conclusion

Information about how the visual system responds to visual motion through the interconnection of behavioral and neural processes has been presented to help advance our understanding of the development of visual perception for prospective control in infancy. Infants show a developmental progress with age as they use visual perceptual information to help guide the execution of anticipatory actions of eye, head, and hand movements. The processing of visual information and the development of object permanence become more efficient around the end of the first year of life. Infants show marked improvements in looming-related brain responses and the ability to switch from a distance or visual-angle strategy to the more efficient time strategy to help tau-guide their reaching movements. With age, infants recognize and differentiate between different radial motions, and show a progression from low- to high-frequency neuronal oscillations during the processing of visual information. Self-produced locomotion experience and the ongoing neural maturational processes may be factors that contribute to the efficiency of visual motion perception during development. Unlike full-terms, preterm infants may have impairments in the functioning of the dorsal visual stream. Impaired functioning of the dorsal stream may contribute to their relatively poorer performances during the processing of visual information. Early detection and identification of preterm infants who could be at risk for developmental problems is thus necessary to help provide early intervention programmes required for their optimal development. When studying the neural correlates of prospective control in infancy, it is of the utmost importance to incorporate behavioral data into EEG analyses to get a better understanding of how the development of brain and behavior is intimately linked.

## Author contributions

SA, FW, and AM have contributed equally to the conception and design of the work and are accountable for all aspects of the work. SA has drafted the work, FW and AM have contributed equally to revising it critically for intellectual content.

## Funding

This project has partly been made possible by the Norwegian ExtraFoundation for Health and Rehabilitation.

### Conflict of interest statement

The authors declare that the research was conducted in the absence of any commercial or financial relationships that could be construed as a potential conflict of interest. The reviewer GR and handling Editor DC declared their shared affiliation, and the handling Editor states that the process nevertheless met the standards of a fair and objective review.
